# The first complete mitochondrial genome for the subfamily Limacodidae and implications for the higher phylogeny of Lepidoptera

**DOI:** 10.1038/srep35878

**Published:** 2016-10-21

**Authors:** Qiu-Ning Liu, Zhao-Zhe Xin, Dan-Dan Bian, Xin-Yue Chai, Chun-Lin Zhou, Bo-Ping Tang

**Affiliations:** 1 Jiangsu Key Laboratory for Bioresources of Saline Soils, Jiangsu Synthetic Innovation Center for Coastal Bio-agriculture, Jiangsu Provincial Key Laboratory of Coastal Wetland Bioresources and Environmental Protection, School of Ocean and Biological Engineering, Yancheng Teachers University, Yancheng 224051, P. R. China

## Abstract

The mitochondrial genome (mitogenome) provides important information for understanding molecular evolution and phylogeny. To determine the systematic status of the family Limacodidae within Lepidoptera, we infer a phylogenetic hypothesis based on the complete mitogenome of *Monema flavescens* (Lepidoptera: Limacodidae). The mitogenome of *M. flavescens* is 15,396 base pairs (bp), and includes 13 protein-coding genes (PCGs), two ribosomal RNA (rRNA) genes, 22 transfer RNA (tRNA) genes, and a control region (CR). The AT skew of this mitogenome is slightly negative and the nucleotide composition is also biased towards A + T nucleotides (80.5%). All PCGs are initiated by ATN codons, except for the cytochrome c oxidase subunit 1 (*cox1*) gene, which is initiated by CGA. All tRNAs display the typical clover-leaf structure characteristic of mitochondrial tRNAs, with the exception of *trnS1* (AGN). The mitogenome CR is 401 bp and consists of several features common to Lepidoptera. Phylogenetic analysis using Bayesian Inference (BI) and Maximum Likelihood (ML) based on nucleotide and amino acid sequences of 13 mitochondrial PCGs indicates that *M. flavescens* belongs to Zygaenoidea. We obtain a well-supported phylogenetic tree consisting of Yponomeutoidea + (Tortricoidea + Zygaenoidea + (Papilionoidea + (Pyraloidea + (Noctuoidea + (Geometroidea + Bombycoidea))))).

The insect mitogenome is a circular molecule 14–19 kilobases in length. It contains 22 tRNAs, 13 PCGs, ATPase subunits 6 and 8 (*atp6* and *atp8*), *cox1-cox3, cytochrome B (cob*), NADH dehydrogenase subunits 1–6 and 4L (*nad1-6* and *nad4L*), the small and large subunit rRNAs (*rrnL* and *rrnS*), and a non-coding element termed the A + T-rich region (CR), which contains initiation sites for transcription and replication^1^,^2^. Because of their unique features, including coding content conservation, maternal inheritance, and rapid evolution, mitogenomes have been informative in diverse studies of molecular evolution, such as phylogenetics, population genetics, and comparative and evolutionary genomics^3^,^4^.

Recent advances in sequencing technologies have led to the rapid increase in mitogenomic data in Genbank, including Lepidopteran mitogenomes. Lepidoptera is the second largest order of insects, accounting for more than 160,000 species^5^. Zygaenidae is a species-rich superfamily of predominantly diurnal moths with a worldwide distribution. This family is particularly diverse in tropical and subtropical Asia and the Palaearctic region^6^. Because of the broad geographical distribution of species, extensive variation in coloration patterns, and an intriguing chemical defence system, Zygaenidae is of great interest to lepidopterists and evolutionary biologists^7^. To date, more than 200 complete or near-complete Lepidopteran mitogenomes are available. However, only one mitogenome of Zygaenoidea has been sequenced^8^. *Monema flavescens* Walker, 1855 is a moth of the Limacodidae family found in Korea, Japan, China, and the Russian Far East. The mitogenome of *M. flavescens* has not been sequenced^9^.

A better understanding of the Lepidopteran mitogenome requires an expansion of taxon and genome samplings. In this study, we sequence and describe the complete mitogenome of *M. flavescens*. We reconstruct a phylogenetic tree based on PCG sequences in order to analyse the evolutionary relationships among Lepidopteran insects. The assembly and annotation of the *M. flavescens* mitogenome will further the study of Zygaenidea mitochondrial genome architecture and phylogenetics. Furthermore, characterization of the *M. flavescens* mitogenome may provide novel insights into the mechanisms underlying mitogenome evolution.

## Methods

### DNA Extraction

The moths of *M. flavescens* were collected in Yancheng, Jiangsu Province. Total DNA was isolated using the Genomic DNA Extraction Kit (SangonBiotech, China) according to the manufacturer’s instructions. Extracted DNA was used to amplify the complete mitogenome by PCR.

### PCR Amplification and Sequencing

For amplification of the *M. flavescens* mitogenome, primer sets were designed based upon mitogenomic sequences obtained from other Lepidopteran insects^10^,^11^. PCR was performed under the following conditions: 3 min at 94 °C, followed by 35 cycles of 30 s at 94 °C, 1–3 min at 48–60 °C, and 10 min at 72 °C. All amplifications were performed on an Eppendorf Mastercycler and Mastercycler gradient in 50 μL reaction volumes. The PCR products were separated by agarose gel electrophoresis (1% w/v) and purified using a DNA gel extraction kit (Transgene, China). The purified PCR products were ligated into the T-vector (SangonBiotech, China) and sequenced at least three times.

### Sequence Assembly and Gene Annotation

Sequence annotation was performed using NCBI BLAST (http://blast.ncbi.nlm.nih.gov/Blast) and the DNAStar package (DNAStar Inc. Madison, WI, USA). The identity of tRNA genes was verified using the tRNAscan-SE program (http://lowelab.ucsc.edu/tRNAscan-SE/)^12^. The nucleotide sequences of PCGs were translated with the invertebrate mitogenome genetic code. Alignments of *M. flavescens* PCGs with various Lepidopteran mitogenomes were performed using Clustal X^13^. Composition skewness was calculated according to the following formulas: AT skew = [A − T]/[A + T]; GC ske = [G−C]/[G + C]. Codon usage was calculated using MEGA version 6.06. Tandem repeats in the A + T-rich region were predicted using the Tandem Repeats Finder program (http://tandem.bu.edu/trf/trf.html)^14^.

### Phylogenetic Analysis

To reconstruct the phylogenetic relationships among Lepidopteran insects, the complete mitogenomes of Lepidopteran species were obtained from GenBank (Table 1). The amino acid sequences for each of the 13 mitochondrial PCGs were aligned using default settings and concatenated. This concatenated set of amino acid and nucleotide sequences was used for phylogenetic analysis, which was performed with the Bayesian inference (BI) and Maximum Likelihood (ML) methods using MrBayes v 3.2.2^15^ and raxmlGUI, respectively. Alignments of individual genes were performed using MAFFT^16^. Gblocks was used to identify conserved regions and remove unreliably aligned sequences within the datasets^17^. For the BI and ML analyses, GTR + I + G was the appropriate model for the nucleotide sequences using MrModeltest 2.3 based on Akaike’s information criterion (AIC)^18^. MtArt + I + G + F was the appropriate model for the amino acid sequence dataset according to ProtTest 3.4 based on AIC^19^. Four independent runs were conducted for 10,000,000 generations, and each was sampled every 1,000 generations. All analyses converged within 10,000,000 generations. We assessed the credibility of the results in two ways. First, the average standard deviation of split frequencies was less than 0.05 in the process of Bayesian. Second, we observed sufficient parameter sampling using software Tracer v1.6. The value of ESS was more than 200. This cumulatively suggested that our data was convergent. Posterior probabilities over 0.95 were interpreted as strongly supported. The mitogenomes of Hepialoidea insects were used as outgroups. The resulting phylogenetic trees were visualized in FigTree v1.4.2.

## Results and Discussion

### Genome Organization and Base Composition

**Figure 1:**
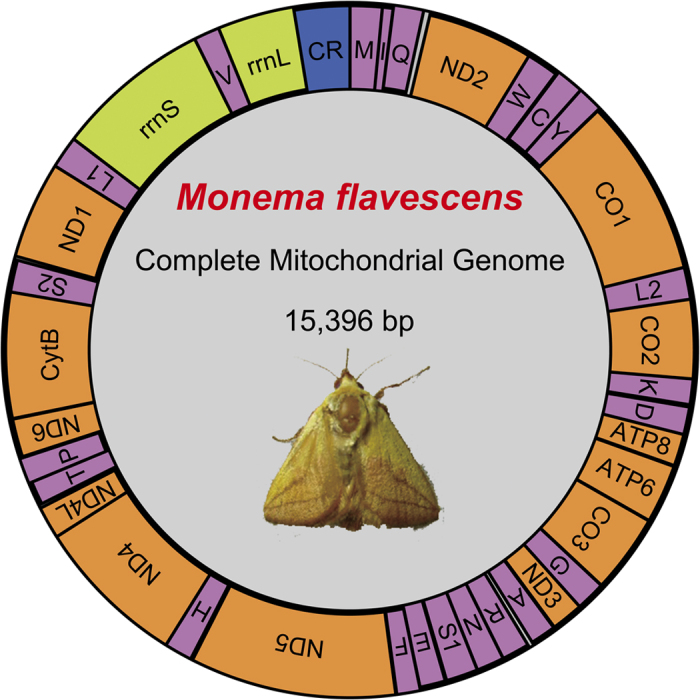
Circular map of the mitogenome of *M. flavescens*. The tRNA genes are labelled according to the IUPAC-IUB. Single-letter amino acids above the bar indicate coding sequence on the major strand, whereas amino acids listed below the bar indicate coding sequence on the minor strand. The one-letter symbols S1, S2, L1, and L2 denote codons *trnS1*(AGN), *trnS2*(UCN), *trnL1*(CUN), and *trnL2*(UUR), respectively.

The mitogenome of *M. flavescens* is a closed circular molecule 15,396 bp in size. The gene content is typical of other Lepidopteran insect mitogenomes, including 22 tRNA genes (one for each amino acid and two each for leucine and serine), 13 PCGs (*cox1-3, nad1-6, nad4L, cob, atp6,* and *atp8*), two mitochondrial rRNA genes (*rrnS* and *rrnL*), and a major non-coding region known as the CR. The majority strand (J strand) encodes 23 genes, while the opposite (N) strand encodes 14 genes (Fig. 1, Table 2). The arrangement of the genes within Lepidopteran mitogenomes is usually highly conserved. While the order and orientation of genes in the *M. flavescens* mitogenome are identical to the only other Zygaenoidea insect sequenced to date, this gene order differs from ancestral insects. Specifically, the placement of the *trnM* gene between the CR and *trnI* in the *M. flavescens* mitogenome (CR, *trnM, trnI, trnQ, nad2*) differs from ancestral insects in which *trnM* is located between *trnQ* and *nad2* (CR, *trnI, trnQ, trnM, nad2*)^20^. However, the ancestral arrangement of the *trnM* gene cluster was also found in ghost moths^21^. This result in *M. flavescens* supports the hypothesis that the ancestral arrangement of the *trnM* gene cluster underwent rearrangement after Hepialoidea diverged from other Lepidopteran lineages. The tRNA gene rearrangements are commonly considered to be a consequence of tandem duplication in a portion of the mitogenome, followed by random or non-random loss of the duplicated copies^22^.

### Skewness, Overlapping, and Intergenic Spacer Regions

The mitogenome of *M. flavescens* has a 29 bp overlap between genes in six locations, with the longest 9 bp overlap located in between *trnW* and *trnC*. The mitogenome of *M. flavescens* contains 167 bp of intergenic spacer sequence spread over 17 regions, ranging in size from 1 to 50 bp (Table 2). The longest spacer sequence is 50 bp located between the *trnQ* and *nad2* genes, and it is extremely A + T rich. The nucleotide composition of the *M. flavescens* mitogenome is as follows: A = 6,275 (40.8%), T = 6,115 (39.7%), G = 1,164 (7.5%), and C = 1,842 (12.0%). As observed in other Lepidopterans, the nucleotide composition of the *M. flavescens* mitogenome is A + T rich (80.5%). This enrichment is lower than in other species, such as *D. punctiferalis* (80.6%), *M. vitrata* (80.7%), *M. testulalis* (80.8%), *L. haraldusalis* (81.5%), and *T. hypsalis* and *N. noctuella* (both 81.4%). In contrast, this enrichment is slightly higher compared to *S. incertulas* (77.1%), *C. suppressalis* (79.7%), and *D. saccharalis* (80.0%). Additionally, the AT skew for the *M. flavescens* mitogenome is slightly positive, indicating a higher occurrence of A compared to T nucleotides. The GC skew values for the *M. flavescens* mitogenome are negative, indicating a higher content of C compared to G nucleotides. This is similar to GC skew values observed in all sequenced Lepidopteran mitogenomes to date.

### Protein-Coding Genes

**Figure 2:**
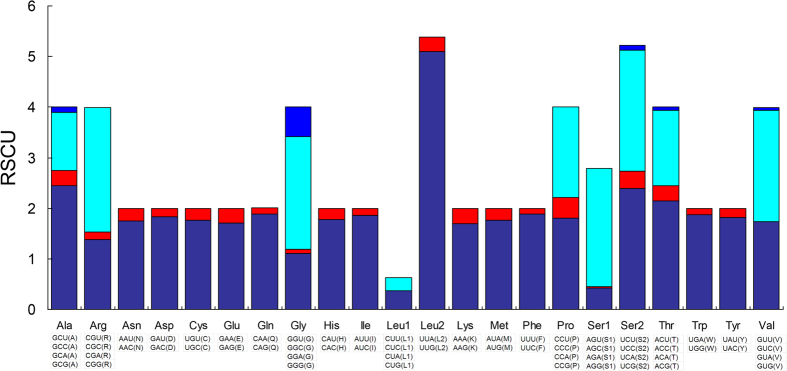
The relative synonymous codon usage (RSCU) in the mitogenome of *M. flavescens*.

The start and stop codons of the 13 PCGs in the *M. flavescens* mitogenome are shown in Table 2. Like invertebrate mitogenomes, 12 of these PCGs begin with the standard ATN start codon, except for *cox1*. Sequence alignment revealed that the open reading frame of *cox1* starts with a CGA codon, which encodes arginine. The putative start codon CGA is common in insects^10^,^23^,^24^. An unusual start codon for the *cox1* gene has also been described in various arthropods^25^,^26^,^27^. In the *M. flavescens* mitogenome, the canonical termination codon, TAA, occurs in seven PCGs. However, the *nad4L* gene utilizes A and the *cox1, cox2, nad2, nad4*, and *cob* genes utilize T as a truncated stop codon instead. Similar results have also been found in other animal mitochondrial genes^28^,^29^,^30^,^31^. Relative synonymous codon usage values for the *M. flavescens* mitogenome are summarized in Table 3 and Fig. 2. The total number of codons in PCGs is 3,716, and the codons CUC, GUC, CCG, UGG, CGG, and AGG are not represented. The most common amino acids in mitochondrial proteins are leucine 2 (Leu 2, 484), isoleucine (Ile, 455), and phenylalanine (Phe, 393), which are likewise highly abundant in mitochondrial proteins in other animals^32^,^33^,^34^. The average AT content of the 13 PCGs is 78.7%. Furthermore, the AT skew of these PCGs is slightly positive, while the GC skew is slightly negative (Table 4).

### Transfer RNA Genes and Ribosomal RNA Genes

**Figure 3:**
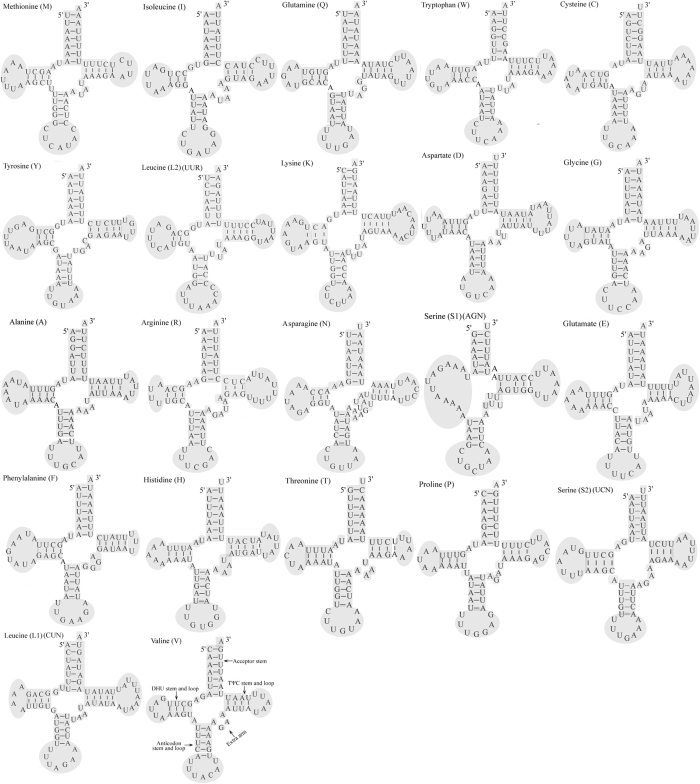
Predicted secondary structures for the tRNA genes in the *M. flavescens* mitogenome.

The tRNAscan-SE Search Server was used to predict the structure of the 22 tRNAs present in the *M. flavescens* mitogenome. Eight tRNAs are encoded by the L-strand and the remaining 14 are encoded by the H-strand. This tRNA genomic architecture is identical to that found in all Lepidopteran species examined to date. Furthermore, all *M. flavescens* tRNAs display the typical clover-leaf secondary structure observed in most mitochondrial tRNAs with the exception of the *trnS1 (AGN*) gene. Interestingly, *trnS1 (AGN*) lacking a stable dihydrouridine arm has been observed in several insects, including Lepidopteran species and metazoan mitogenomes^35^,^36^,^37^,^38^. A 7 bp amino acid acceptor stem, in addition to the anticodon stem and loop (7 bp), are both conserved in all tRNAs. While a total of 25 unmatched base pairs were detected in these tRNAs (Fig. 3), 18 of them are G-U pairs, which form a weak bond and are well-known non-canonical pairs in tRNA secondary structures. The remaining seven mismatches include one C-U and six U-U pairs. 22 tRNAs in the *M. flavescens* mitogenome are 1,513 bp long, each of which range in size from 63 to 73 bp. The A + T content is 82.4%. The AT skew for both tRNAs and rRNAs is slightly positive, indicating a higher occurrence of A compared to T nucleotides. The GC skew for both tRNAs and rRNAs is slightly negative, indicating a higher occurrence of C compared to G nucleotides. The two rRNA genes (*rrnS* and *rrnL*) present in *M. flavescens* mitogenome are located between *trnL1* (CUN) and *trnV* or between *trnV* and the A + T-rich region, respectively. The sizes of *rrnL* and *rrnS* are 1,359 bp and 792 bp, respectively. The A + T content of the two *rRNAs* is 84.5% (Table 4).

### Control Region

**Figure 4:**
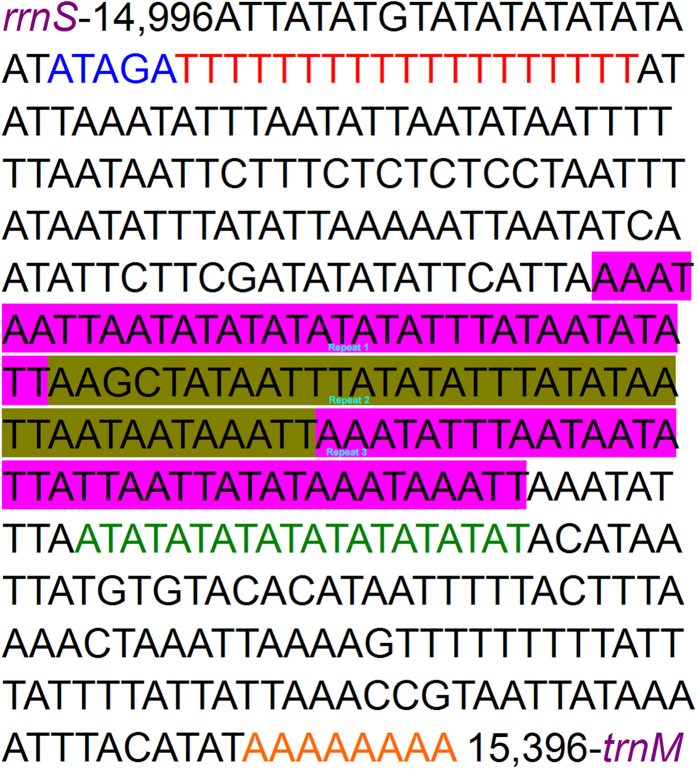
Features present in the A + T-rich region of the *M. flavescens* mitogenome. The reverse strand sequence is shown. Coloured nucleotides indicate the ATATG motif (red), the poly-T stretch (blue), two microsatellite T/A repeat sequences (green), and the poly-A stretch (pink). Two tandem repeats 51 bp in length are indicated in red and black single underline.

The CR possesses essential elements involved in the initiation of replication and transcription of the mitogenome^39^. The CR of the *M. flavescens* mitogenome extends over 401 bp and is located between *rrnS* and *trnM*. The CR contains the highest A + T content (93.3%) in the entire mitogenome. Both the AT skew and GC skew for the CR are slightly negative, indicating that T and C are more abundant than A and G, respectively. Several conserved structures found in other Lepidopteran mitogenomes are also observed in the A + T-rich region of *M. flavescens*. This includes the motif ‘ATAGA’ and a poly-T stretch downstream of *rrnS*, which is widely conserved in Lepidopteran mitogenomes and may represent the origin of minority or light strand replication^40^. A poly-A commonly observed in other Lepidopteran mitogenomes is also found immediately upstream of the *trnM* gene. We identified microsatellite (AT)_10_ elements in the A + T-rich region. Multiple tandem repeat elements are typically present in the A + T-rich region of most insects. However, only three tandem repeats are found in the CR of the *M. flavescens* mitogenome (Fig. 4).

### Phylogenetich Analyses

**Figure 5:**
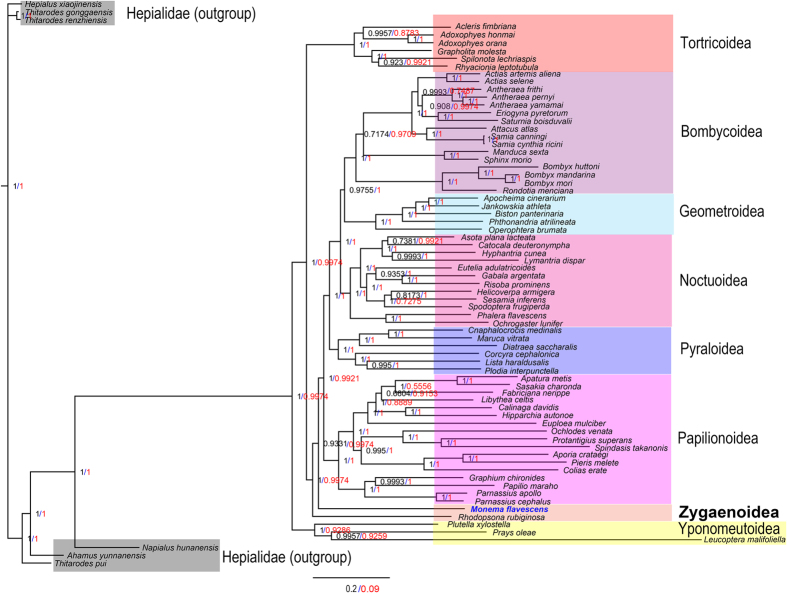
Phylogenetic trees inferred from amino acid (red) and nucleotide (black) sequences of 13 PCGs of the mitogenome using BI analysis.

**Figure 6:**
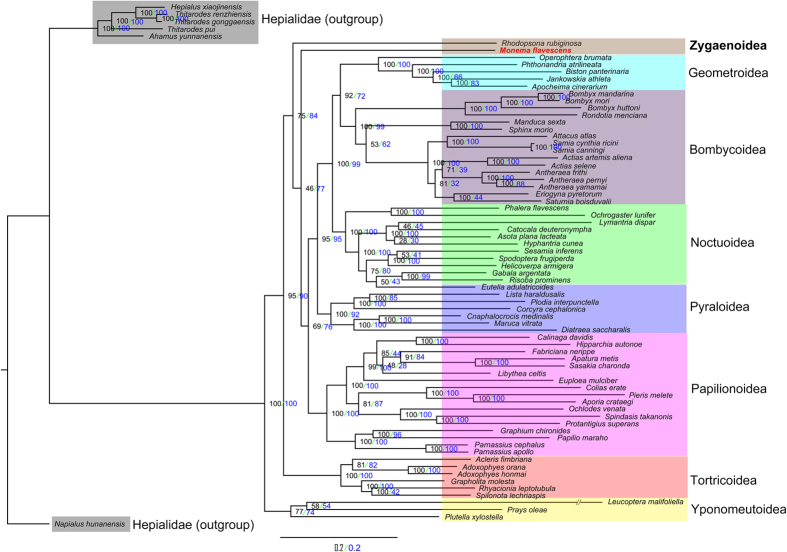
Phylogenetic trees inferred from amino acid (blue) and nucleotide (black) sequences of 13 PCGs of the mitogenome using ML analysis.

Phylogenetic relationships within the Zygaenoidea superfamily are highly debated. In the present study, concatenated amino acid and nucleotide sequences of the 13 PCGs from mitogenomes obtained from nine Lepidopteran superfamilies are used to reconstruct phylogenetic relationships by the BI and ML methods (Figs 5 and 6). The monophyly of each superfamily is generally well supported. The best-supported phylogenetic relationship found in this study is as follows: Yponomeutoidea + (Tortricoidea + Zygaenoidea + (Papilionoidea + (Pyraloidea + (Noctuoidea + (Geometroidea + Bombycoidea))))). The analyses show that *M. flavescens* belongs in the Zygaenoidea superfamily. Both Papilionoidea and Tortricoidea superfamilies are most closely related to Zygaenoidea. More mitogenomes from Zygaenoidea insects were required to resolve the position of Zygaenoidea and the relationships among these superfamilies. Our phylogeny clearly separates and demonstrates a similar topology as that derived from traditional classifications and other molecular data^41^,^42^.

## Additional Information

****Accession codes:**** The *M. flavescens* mitogenome was submitted under the accession number KU946971 to NCBI.

**How to cite this article**: Liu, Q.-N. *et al*. The first complete mitochondrial genome for the subfamily Limacodidae and implications for the higher phylogeny of Lepidoptera. *Sci. Rep.* **6**, 35878; doi: 10.1038/srep35878 (2016).

## Figures and Tables

**Table 1:** List of the complete mitogenomes of Lepidopteran insects.

Superfamily	Family	Subfamily	Species	Genbank No.
Zygaenoidea	Limacodidae		*Monema flavescens*	KU946971
Zygaenoidea	Zygaenidae	Chalcosiinae	*Rhodopsona rubiginosa*	KM244668
Bombycoidea	Bombycidae	Bombycinae	*Bombyx mandarina*	AB070263
Bombycoidea	Bombycidae	Bombycinae	*Bombyx mori*	AF149768
Bombycoidea	Bombycidae	Bombycinae	*Bombyx huttoni*	KP216766
Bombycoidea	Bombycidae	Bombycinae	*Rondotia menciana*	KC881286
Bombycoidea	Saturniidae	Saturniinae	*Samia cynthia ricini*	JN215366
Bombycoidea	Saturniidae	Saturniinae	*Actias selene*	JX186589
Bombycoidea	Saturniidae	Saturniinae	*Antheraea pernyi*	AY242996
Bombycoidea	Saturniidae	Saturniinae	*Antheraea yamamai*	EU726630
Bombycoidea	Saturniidae	Saturniinae	*Eriogyna pyretorum*	FJ685653
Bombycoidea	Saturniidae	Saturniinae	*Saturnia boisduvalii*	EF622227
Bombycoidea	Sphingidae	Sphinginae	*Manduca sexta*	EU286785
Bombycoidea	Sphingidae	Sphinginae	*Sphinx morio*	KC470083
Bombycoidea	Saturniidae	Saturniinae	*Antheraea frithi*	KJ740437
Bombycoidea	Saturniidae	Saturniinae	*Attacus atlas*	KF006326
Bombycoidea	Saturniidae	Saturniinae	*Actias artemis aliena*	KF927042
Bombycoidea	Saturniidae	Saturniinae	*Samia canningi*	KJ159909
Geometroidea	Geometridae	Ennominae	*Biston panterinaria*	JX406146
Geometroidea	Geometridae	Ennominae	*Phthonandria atrilineata*	EU569764
Geometroidea	Geometridae	Ennominae	*Jankowskia athleta*	KR822683
Geometroidea	Geometridae	Ennominae	*Apocheima cinerarium*	KF836545
Geometroidea	Geometridae	Larentiinae	*Operophtera brumata*	KP027400
Hepialoidea	Hepialidae		*Ahamus yunnanensis*	HM744695
Hepialoidea	Hepialidae		*Thitarodes renzhiensis*	HM744694
Hepialoidea	Hepialidae		*Hepialus xiaojinensis*	KT834973
Hepialoidea	Hepialidae		*Thitarodes pui*	KF908880
Hepialoidea	Hepialidae		*Thitarodes gonggaensis*	KP718817
Hepialoidea	Hepialidae		*Napialus hunanensis*	KJ632465
Noctuoidea	Erebidae	Lymantriinae	*Lymantria dispar*	FJ617240
Noctuoidea	Erebidae	Arctiinae	*Hyphantria cunea*	GU592049
Noctuoidea	Erebidae	Aganainae	*Asota plana lacteata*	KJ173908
Noctuoidea	Erebidae	Erebinae	*Catocala deuteronympha*	KJ432280
Noctuoidea	Euteliidae	Euteliinae	*Eutelia adulatricoides*	KJ185131
Noctuoidea	Nolidae	Chloephorinae	*Gabala argentata*	KJ410747
Noctuoidea	Nolidae	Risobinae	*Risoba prominens*	KJ396197
Noctuoidea	Noctuidae	Amphipyrinae	*Sesamia inferens*	JN039362
Noctuoidea	Noctuidae	Amphipyrinae	*Spodoptera frugiperda*	KM362176
Noctuoidea	Noctuidae	Heliothinae	*Helicoverpa armigera*	GU188273
Noctuoidea	Notodontidae	Phalerinae	*Phalera flavescens*	JF440342
Noctuoidea	Notodontidae	Thaumetopoeinae	*Ochrogaster lunifer*	AM946601
Papilionoidea	Hesperiidae	Hesperiinae	*Ochlodes venata*	HM243593
Papilionoidea	Lycaenidae	Aphnaeinae	*Spindasis takanonis*	HQ184266
Papilionoidea	Lycaenidae	Theclinae	*Protantigius superans*	HQ184265
Papilionoidea	Nymphalidae	Apaturinae	*Apatura metis*	JF801742
Papilionoidea	Nymphalidae	Apaturinae	*Sasakia charonda*	AP011824
Papilionoidea	Nymphalidae	Calinaginae	*Calinaga davidis*	HQ658143
Papilionoidea	Nymphalidae	Danainae	*Euploea mulciber*	HQ378507
Papilionoidea	Nymphalidae	Heliconiinae	*Fabriciana nerippe*	JF504707
Papilionoidea	Nymphalidae	Libytheinae	*Libythea celtis*	HQ378508
Papilionoidea	Nymphalidae	Satyrinae	*Hipparchia autonoe*	GQ868707
Papilionoidea	Papilionidae	Papilioninae	*Graphium chironides*	KP159289
Papilionoidea	Papilionidae	Papilioninae	*Papilio maraho*	FJ810212
Papilionoidea	Papilionidae	Parnassiinae	*Parnassius apollo*	KF746065
Papilionoidea	Papilionidae	Parnassiinae	*Parnassius cephalus*	KP100655
Papilionoidea	Pieridae	Pierinae	*Aporia crataegi*	JN796473
Papilionoidea	Pieridae	Pierinae	*Pieris melete*	EU597124
Papilionoidea	Pieridae	Coliadinae	*Colias erate*	KP715146
Pyraloidea	Crambidae	Spilomelinae	*Maruca vitrata*	KJ466365
Pyraloidea	Crambidae	Crambinae	*Diatraea saccharalis*	FJ240227
Pyraloidea	Crambidae	Pyraustinae	*Cnaphalocrocis medinalis*	JN246082
Pyraloidea	Pyralidae	Galleriinae	*Corcyra cephalonica*	HQ897685
Pyraloidea	Pyralidae	Phycitinae	*Plodia interpunctella*	KP729178
Pyraloidea	Pyralidae	Epipaschiinae	*Lista haraldusalis*	KF709449
Tortricoidea	Tortricidae	Olethreutinae	*Rhyacionia leptotubula*	JX028270
Tortricoidea	Tortricidae	Olethreutinae	*Spilonota lechriaspis*	HM204705
Tortricoidea	Tortricidae	Olethreutinae	*Grapholita molesta*	HQ392511
Tortricoidea	Tortricidae	Tortricinae	*Adoxophyes orana*	JX872403
Tortricoidea	Tortricidae	Tortricinae	*Acleris fimbriana*	HQ662522
Tortricoidea	Tortricidae	Tortricinae	*Adoxophyes honmai*	DQ073916
Yponomeutoidea	Lyonetiidae		*Leucoptera malifoliella*	JN790955
Yponomeutoidea	Yponomeutidae	Praydinae	*Prays oleae*	KM874804
Yponomeutoidea	Plutellidae		*Plutella xylostella*	JF911819

**Table 2:** Summary of the mitogenome of *M. flavescens*.

Gene	Direction	Location	Size	Anticodon	Start codon	Stop codon	Intergenic nucleotides
*trnM*	F	1–68	68	CAT	—	—	2
*trnI*	F	71–135	65	GAT	—	—	−3
*trnQ*	R	133–201	69	TTG	—	—	50
*nad2*	F	252–1266	1015	—	ATT	T	−3
*trnW*	F	1264–1332	69	TCA	—	—	−9
*trnC*	R	1324–1388	65	GCA	—	—	5
*trnY*	R	1394–1462	67	GTA	—	—	3
*cox1*	F	1466–2996	1531	—	CGA	T	0
*trnL2(UUR)*	F	2997–3065	69	TAA	—	—	0
*cox2*	F	3066–3747	682	—	ATG	T	0
*trnK*	F	3748–3820	73	CTT	—	—	16
*trnD*	F	3837–3899	63	GTC	—	—	0
*atp8*	F	3900–4061	162	—	ATT	TAA	−7
*atp6*	F	4055–4728	674	—	ATG	TAA	3
*cox3*	F	4732–5517	786	—	ATG	TAA	2
*trnG*	F	5520–5588	69	TCC	—	—	0
*nad3*	F	5589–5942	354	—	ATT	TAA	6
*trnA*	F	5949–6018	70	TGC	—	—	36
*trnR*	F	6055–6121	67	TCG	—	—	1
*trnN*	F	6123–6196	74	GTT	—	—	1
*trnS1(AGN)*	F	6198–6263	66	GCT	—	—	10
*trnE*	F	6274–6343	70	TTC	—	—	−2
*trnF*	R	6342–6410	69	GAA	—	—	7
*nad5*	R	6418–8136	1719	—	ATC	TAA	15
*trnH*	R	8152–8219	68	GTG	—	—	0
*nad4*	R	8220–9555	1336	—	ATG	T	0
*nad4L*	R	9556–9839	284	—	ATA	A	24
*trnT*	F	9864–9927	64	TGT	—	—	0
*trnP*	R	9928–9993	66	TGG	—	—	8
*nad6*	F	10,002–10,520	519	—	ATA	TAA	4
*cob*	F	10,525–11,674	1150	—	ATG	T	0
*trnS2(UCN)*	F	11,675–11,744	70	TGA	—	—	−5
*nad1*	R	11,770–12,708	939	—	ATG	TAA	0
*trnL1(CUN)*	R	12,709–12,779	71	TAG	—	—	0
*rrnL*	R	12,780–14,138	1359	—	—	—	0
*trnV*	R	14,139–14,203	65	TAC	—	—	0
*rrnS*	R	14,204–14,995	792	—	—	—	0
A + T-rich region		14,996–15,396	401	—	—	—	—

**Table 3:** Codon number and RSCU in *M. flavescens* mitochondrial PCGs.

Codon	Count	RSCU	Codon	Count	RSCU	Codon	Count	RSCU	Codon	Count	RSCU
UUU(F)	370	1.88	UCU(S2)	99	2.39	UAU(Y)	153	1.82	UGU(C)	29	1.76
UUC(F)	23	0.12	UCC(S2)	14	0.34	UAC(Y)	15	0.18	UGC(C)	4	0.24
UUA(L2)	459	5.1	UCA(S2)	99	2.39	UAA(*)	8	2	UGA(W)	89	1.87
UUG(L2)	25	0.28	UCG(S2)	4	0.1	UAG(*)	0	0	UGG(W)	6	0.13
CUU(L1)	33	0.37	CCU(P)	57	1.81	CAU(H)	62	1.77	CGU(R)	18	1.38
CUC(L1)	0	0	CCC(P)	13	0.41	CAC(H)	8	0.23	CGC(R)	2	0.15
CUA(L1)	23	0.26	CCA(P)	56	1.78	CAA(Q)	60	1.88	CGA(R)	32	2.46
CUG(L1)	0	0	CCG(P)	0	0	CAG(Q)	4	0.13	CGG(R)	0	0
AUU(I)	424	1.86	ACU(T)	76	2.14	AAU(N)	225	1.75	AGU(S1)	18	0.43
AUC(I)	31	0.14	ACC(T)	11	0.31	AAC(N)	32	0.25	AGC(S1)	1	0.02
AUA(M)	238	1.76	ACA(T)	53	1.49	AAA(K)	93	1.69	AGA(S1)	97	2.34
AUG(M)	32	0.24	ACG(T)	2	0.06	AAG(K)	17	0.31	AGG(S1)	0	0
GUU(V)	64	1.74	GCU(A)	72	2.44	GAU(D)	55	1.83	GGU(G)	55	1.11
GUC(V)	0	0	GCC(A)	9	0.31	GAC(D)	5	0.17	GGC(G)	4	0.08
GUA(V)	81	2.2	GCA(A)	34	1.15	GAA(E)	66	1.71	GGA(G)	111	2.23
GUG(V)	2	0.05	GCG(A)	3	0.1	GAG(E)	11	0.29	GGG(G)	29	0.58

A total of 3,716 codons were analysed, excluding the initiation and termination codons. Amino acids encoded by these codons are labelled according to the IUPAC-IUB single-letter amino acid codes.

**Table 4:** Composition and skewness in the *M. flavescens* mitogenome.

*M. flavescens*	Size (bp)	A%	G%	T%	C%	A + T%	AT skewness	GC skewness
mitogenome	15,396	40.8	7.5	39.7	12.0	80.5	0.014	−0.231
PCGs	11,145	39.9	8.5	38.8	12.8	78.7	0.014	−0.202
tRNAs	1513	41.6	7.5	40.8	10.1	82.4	0.010	−0.148
rRNAs	2151	43.3	4.6	41.2	10.9	84.5	0.025	−0.406
control region	401	43.7	2.00	49.6	4.7	93.3	−0.063	−0.403
